# Selection of modalities, prescription, and technical issues in children on peritoneal dialysis

**DOI:** 10.1007/s00467-008-0848-4

**Published:** 2009-08-01

**Authors:** Enrico Verrina, Valeria Cappelli, Francesco Perfumo

**Affiliations:** Dialysis Unit, Nephrology and Dialysis Division, Giannina Gaslini Institute, Largo G. Gaslini, 5, 16148 Genoa, Italy

**Keywords:** Peritoneal dialysis, Children, Automated peritoneal dialysis, Peritoneal membrane function, Peritoneal dialysis solutions, Peritoneal dialysis prescription

## Abstract

Peritoneal dialysis (PD) is widely employed as a dialytic therapy for uraemic children, especially in its automated form (APD), that is associated with less burden of care on patient and family than continuous ambulatory PD. Since APD offers a wide range of treatment options, based on intermittent and continuous regimens, prescription can be individualized according to patient’s age, body size, residual renal function, nutritional intake, and growth-related metabolic needs. Transport capacity of the peritoneal membrane of each individual patient should be assessed, and regularly monitored, by means of standardized peritoneal function tests validated in pediatric patients. To ensure maximum recruitment of peritoneal exchange area, fill volume should be scaled to body surface area and adapted to each patient, according to clinical tolerance and intraperitoneal pressure. PD solutions should be employed according to their biocompatibility and potential ultrafiltration capacity; new pH-neutral, glucose-free solutions can be used in an integrated way in separate dwells, or by appropriately mixing during the same dialytic session. Kinetic modelling software programs may help in the tailoring of PD prescription to individual patients’ characteristics and needs. Owing to advances in the technology of new APD machines, greater programming flexibility, memorized delivery control, and tele-dialysis are currently possible.

## Introduction

Chronic peritoneal dialysis (CPD) currently represents the dialysis treatment modality most commonly prescribed for pediatric patients with end-stage renal disease throughout much of the world, and the preference for CPD over haemodialysis is most pronounced among infants and young children 0 to 5 years of age [1–3].

In this study we addressed the issues of CPD modality selection and prescription, including some technical aspects of the modalities; for reason of space, an effort has been made to summarize accurately current knowledge specific to these issues, but readers are encouraged to consult references cited throughout the paper for more details on specific topics.

### Selection of peritoneal dialysis modality

The aim of the process of CPD modality selection and prescription is to tailor the treatment schedule to the needs of each individual patient, according to a series of parameters such as the patient’s age, body size, associated non-renal diseases, residual renal function (RRF), clinical conditions, blood pressure, nutritional status, and transport characteristics of the peritoneal membrane (PM). At the same time, potential negative effects of CPD treatment on the patient’s metabolism and on PM viability should be taken into account. Finally, the burden of CPD treatment should be compatible with a satisfactory level of psychological and social rehabilitation of the patient and of his/her family. During the past 10–15 years, several technical improvements in materials and devices for peritoneal dialysis (PD), the development of more biocompatible PD solutions, and the employment of computer technology, have provided dialysis staff with valuable tools to improve the efficacy and tolerability of CPD treatment and also to check that the treatment dose has been delivered.

Since the selection of the most proper CPD modality, as well as the prescription of the optimal treatment schedule, should be based on an accurate assessment of the PM transport characteristics of individual patients, the issue of PM function tests will be addressed first, followed by a brief description of conventional and new PD solutions. Then, CPD treatment methods and regimens will be considered, together with the prescription tools that are currently available.

## Peritoneal membrane function tests

Peritoneal solute and fluid transport may vary considerably from patient to patient and in the same patient during different phases of CPD treatment, as a consequence of the recurrence and/or severity of peritonitis episodes or of the exposure of the PM to CPD solutions and materials. Therefore, PM transport characteristics should be assessed at the beginning of CPD (usually a month after the patient has started peritoneal dialysis) and then monitored every 6–12 months and during recurrent or particularly severe peritonitis or any other clinical event that may cause changes in transport capacity [4, 5].

The application of PM function tests to paediatric patients has long been hampered by a lack of standardization of dialysis mechanics during the test. Appropriate scaling for body size plays a central role for this standardization and for the calculation of membrane function parameters. While in infants the peritoneal surface area per unit body weight (BW) is twice that of adults, the relationship between body surface area (BSA) and PM surface area is constant and age independent. In early pediatric transport studies, standardization of exchange volumes by BW led to a false perception of differences in peritoneal permeability between children and adults, with an enhanced transport function in the youngest patients that was due to faster solute equilibration associated with the use of relatively small dwell volumes [6]. On the contrary, scaling the exchange volume by BSA maintains the relationship between dialysate volume and PM surface area across populations, and makes comparison of peritoneal transport properties between patients of different body sizes possible [7, 8]. An exchange volume of 1,100 ml/m^2^ BSA approximates the standard BSA-based volume of 2,000 ml/1.73 m^2^ applied to adult patients.

### Measurement of mass transfer area coefficient

Diffusive permeability of the peritoneal membrane can be expressed by means of the mass transfer area coefficient (MTAC), which describes the maximum clearance theoretically achievable at a constantly maximum gradient for diffusion (i.e. when dialysate solute concentration is zero) and is independent of dialysate glucose concentration. MTAC can be calculated with the help of computer technology, which can give reliable results also in pediatric patients [9]. Comparison of MTAC values obtained in patients of different ages is possible if exchange volume has been standardized to BSA [9, 10]. However, relatively greater solute transport capacity has been reported in infants, as a consequence of higher peritoneal permeability or larger effective surface area of the peritoneal membrane [10].

### The peritoneal equilibration test

This test remains the most commonly employed means of characterizing PM transport capacity in adults as well as in children [10–13]. Urea and creatinine dialysate-to-plasma (D/P) ratios and dialysate glucose concentration to initial dialysate glucose concentration at time 0 (D/D0), calculated at 2 h and 4 h of a standard peritoneal equilibration test (PET) conducted with a 1,100 ml/m^2^ dwell volume of a 2.5% dextrose PD solution, can be compared to the results from a large paediatric study in which the same PET procedure was adopted [10]. Thus, patients will be characterized as having high, high average, low average or low, solute transport (Table 1). Similarly to what has been reported in adult patients, the high transporter status may be associated with poor treatment outcome and has been identified as a significant risk factor for inadequate weight control, poor statural growth [14], and low-turnover bone disease [15].

**Table 1:** Peritoneal equilibration test results for urea, creatinine and glucose. The four categories of peritoneal transport are bordered by the maximum, mean + 1 standard deviation (SD), mean, mean −1 SD, and minimum values for the study population. *D/P* dialysate-to-plasma ratio, *D/D0* dialysate glucose to initial dialysate glucose concentration ratio. Data adapted from [10] and used with permission

Category of peritoneal transport	D/P urea^a^	D/P creatinine^a^	D/D0 glucose^a^
High	0.91–0.94	0.77–0.88	0.12–0.21
High average	0.82–0.90	0.64–0.76	0.22–0.32
Low average	0.74–0.81	0.51–0.63	0.33–0.42
Low	0.54–0.73	0.37−0.50	0.43–0.55

^a^At a 4 h dwell of an exchange performed with 1,100 ml/m^2^ BSA of a 2.5% dextrose solution

The PET can be also performed with a 4.25% dextrose PD solution to obtain more accurate information on ultrafiltration (UF) capacity and sodium sieving [16].

Recently, Warady and Jennings reported that the PET results obtained at 2 h and 4 h, based on either creatinine or glucose transport in 20 children who had been on PD for 7 months or less, provided identical characterization of peritoneal membrane transport capacity for the same solute [17]. Therefore, the authors proposed the use in paediatric patients of a simplified, 2 h PET procedure, the so-called short PET, as already described in adult patients by Twardowski et al. in the original publication of the PET [11]. Since the short PET is more convenient for patients, families, and nursing staff and is associated with cost savings, the adoption of this procedure may help in the evaluation of transport characteristics of the peritoneal membrane on a more routine basis among paediatric PD centres. However, further study with a larger patient cohort is required to confirm the accuracy of the short PET in the characterization of membrane transport capacity in this setting [18].

Two other tests for peritoneal membrane function that have given reliable results in adults as well as in paediatric patients, but are less frequently employed than the PET in the clinical setting, are the so-called standard permeability analysis and the personal dialysis capacity test.

### Standard permeability analysis

In this test, polydisperse dextran-70 is added to the PD solution employed in the PET so that simultaneous measurement is obtained of transcapillary ultrafiltration (UF), marker clearance rates, and intraperitoneal volume (IPV). Standard permeability analysis (SPA) conducted with a test IPV of 1,200 ml/m^2^ and a 1.36% or 3.86% glucose PD solution gave comparable results in adult and pediatric patients [19, 20].

### Personal dialysis capacity test

This test [21] is based on the three-pore model of solute and fluid transport across the peritoneum, which is characterized as a heteroporous three-pore membrane with few (approximately 1–2%) water-exclusive ultra-small pores, called aquaporins (radius 0.2–0.4 nm), a small percentage (approximately 5%) of large pores (radius 20–30 nm), and a majority (approximately 90–95%) of small pores (radius 4–6 nm). Small solute transport occurs primarily by diffusion across the small pores, while proteins and other macromolecules are driven by convection across the large pores. Fluid transport is determined by crystalloid and colloid osmotic pressures and can occur across all three pathways. By personal dialysis capacity (PDC) test the following three parameters can be calculated: (1) the effective peritoneal surface area, or unrestricted pore area over diffusion distance (A_0/_ΔX), corresponding to the diffusion capacity for solutes; (2) absorption, i.e. the final rate of fluid reabsorption from the abdominal cavity, and (3) the large-pore volume flow, which represents the rate of protein-rich fluid passing through the large pores from the blood to the dialysate. PDC protocol includes five exchanges to be performed in 24 h, using different dwell times and two glucose solutions for patients on continuous ambulatory peritoneal dialysis (CAPD); a simplified protocol for patients on APD is also available [22]. The PDC test has been successfully employed in children to model individual peritoneal membrane function [22]. In one paediatric study, D/P or D/D0 ratios derived from PET analysis were used to estimate A_0/_ΔX with a specific computer program [23].

## Peritoneal dialysis solutions

Several types of PD solutions have been made commercially available that achieve satisfactory removal of fluid and waste products and maintain acid–base and calcium balance and electrolyte homoeostasis. The increasing knowledge of the harmful effects of prolonged exposure of the peritoneal membrane to standard PD solutions with high glucose and lactate concentration, low pH, high osmolarity, and high level of glucose degradation products (GDPs) has led to the development of more biocompatible, second-generation PD solutions. Studies done recently support the hypothesis that peritoneal membrane hyper-vascularization and fibrosis observed during long-term PD are correlated to acute and chronic toxicity of conventional PD fluids. Furthermore, progressive decline of residual renal function, which is considered a major determinant of PD treatment outcome, can be exacerbated by the metabolic and cardiovascular burden related to glucose load, GDPs accumulation, and oxidative stress [24–26].

Glucose remains the most widely employed osmotic agent in the clinical setting. The crystalloid osmotic effect of glucose, which is exerted through the aquaporins, can be effectively enhanced by increasing its concentration in the PD fluid. The rapid and variable absorption rate of glucose from the peritoneal fluid dissipates the osmotic gradient and makes it unsuitable to obtain adequate UF during long dwells and in patients with high peritoneal transport rates. Glucose absorption may worsen the anorexia, hyperglycaemia, dyslipidaemia, and insulin resistance as well as the increased oxidative stress that are often associated with the uraemic syndrome. Moreover, long-term exposure to the elevated glucose concentration of PD fluids contributes to structural (submesothelial thickening and fibrosis, and vascular proliferation) and functional (UF failure) changes in the peritoneal membrane. The main mechanisms by which glucose-based PD fluids induce these deleterious effects on peritoneum are represented by:

- hyperosmolar stress
- the presence of highly reactive glucose GDPs that impair mesothelial cell function and modulate cytokine generation
- glycation of structural proteins and formation of advanced glycation end products (AGEs)
- effects on peritoneal cell metabolism via the polyol pathway, protein kinase activation and gene induction [26–28].

Reduced formation of GDPs has been obtained by the separation of glucose from other contents in a double-chamber bag system that allows glucose sterilization at a lower pH than is possible in single-chamber bags [28, 29]. Significant reduction of plasma AGE levels has been reported in paediatric patients by administration of low-GDP PD solutions [30].

In summary, glucose is effective in UF induction along short dwells, but the lowest glucose concentration of PD solution should be used in daily practice while still being compatible with the patient’s clinical needs [31].

### Icodextrin

As an alternative to glucose-based PD solutions during prolonged dwells, PD solutions containing a polymer of glucose with an average molecular weight (MW) of 16,200 Da (icodextrin) have been extensively studied and applied to adult PD patients. The colloid osmotic effect of a 7.5% icodextrin solution that is exerted through the small pore was able to obtain sustained, net UF during a 14 h dwell [32]. Studies in paediatric patients showed the same UF profile as in adults, an increase in solute removal, and rare, mild side effects (skin rash) [33–35]. By comparing the results of two 4 h PETs, performed on nine paediatric patients and using 3.86% glucose and 7.5% icodextrin as a test solution, Rusthoven et al. [36] found that the two solutions had different effects on the change in intraperitoneal pressure (IPP); during the PET performed with a 3.86% glucose solution, the increase in IPP was positively correlated with transcapillary UF and inversely correlated with the patients’ BSA, while, by using an icodextrin solution, they found that the IPP hardly increased during the 4 h dwell and no correlation was found with fluid kinetics or patient BSA.

Since the colloid osmotic effect exerted by icodextrin does not induce sodium sieving, sodium removal is usually higher than that obtained with glucose-based solutions [37].

In children icodextrin absorption was reported to be 45% over 14 h [34]. Icodextrin is metabolized by amylase to maltose and a number of oligosaccharides, whose serum levels usually reach a steady state within 2 weeks from the start of treatment and go back to zero 2 weeks after discontinuation of the use of icodextrin solution [33]. Sterile peritonitis was reported in some patients treated with icodextrin and was caused by peptidoglycan contamination of the dialysate by thermophilic, acidophilic bacteria [38].

In vitro and ex vivo studies have shown that icodextrin solution is more biocompatible with the peritoneal membrane than is glucose-based solutions, possibly due to its iso-osmolar property, lack of glucose, and lower GDP content [39]. However, it has been recently reported that icodextrin may also inhibit the normal process of mesothelial cell repopulation and induce repair by means of connective tissue formation [40].

In summary, icodextrin solution is indicated for: long night-time dwell in continuous ambulatory PD (CAPD); long daytime dwell in continuous cycling PD (CCPD); patients with type I UF failure or transient UF failure associated with peritonitis. Icodextrin is currently licensed for use in not more than one dwell per day, out of concern for the potential side effects of its low molecular weight metabolites.

### Combination of different solutions

With the aim of optimizing fluid removal and reducing glucose exposure, a combination solution of glucose and icodextrin has been employed with encouraging results during a daytime dwell in adult patients. [41, 42]. Studies on the use of bimodal PD solutions in paediatric patients should be encouraged.

The concept of prescribing a mixture of dialysis fluids in order to take the maximum advantage of each component has been applied to the use of amino acid (AA) PD solutions. In children on CAPD the effect on nutritional status of using an AA solution in a long dwell was anecdotal. Increases in blood urea nitrogen and worsening of acidosis have been observed [43]. On the other hand, combined intraperitoneal infusion of AA and glucose during nocturnal APD sessions promoted the utilization of AA for protein synthesis [44]. This schedule was reported to improve anthropometric parameters in children on APD [45] and to influence positively muscle protein turnover in adult patients [46].

A 1.1% AA solution is as osmotically efficient as a 1.36% glucose solution is; moreover, a certain increase in solute removal and UF may be expected, since AAs tend to induce peritoneal vasodilatation and, hence, recruitment of microvascular surface area to a greater extent than glucose does [47].

In summary, the use of AA solution in children on APD can be indicated in order to improve treatment biocompatibility and to supply AA in malnourished patients. However, in these patients enteral nutrition should be used whenever possible to improve nutritional status [31].

### Bicarbonate-based PD solutions

The use of lactate-buffered PD solutions with low pH is associated with a series of well-known clinical, metabolic and biocompatibility drawbacks (Table 2). Neutral pH (7.0–7.6) PD solutions containing, 34 mmol/l of bicarbonate, or 25 mmol/l of bicarbonate plus 15 mmol/l of lactate, are commercially available in multi-compartment bag systems [31]. Adult and paediatric studies have shown that the use of these bicarbonate-buffered PD solutions is associated with better biocompatibility, more effective correction of acidosis, and lower incidence of infusion pain than that of conventional lactate-buffered solutions [48–50]. Schmitt et al. [51] found that peritoneal mass transfer kinetics were similar with bicarbonate and lactate for water and most solutes, except for slightly lower phosphate and creatinine transport rates, at 1 h dwell time with bicarbonate solutions. These more physiological PD fluids have been shown to prevent hyperperfusion and to reduce the loss of proteins into dialysate; their use has been associated with lower intraperitoneal pressure, reflecting enhanced tolerance of fill volume, but also with a reduction of the unrestricted area over diffusion distance and of the vascular exchange area [50].

**Table 2:** Effects of toxins on membrane integrity (*left side of table*) and clinical and metabolic drawbacks (*right side of table*) that may be correlated with the use of lactate-buffered PD solutions

Effects of toxins on membrane integrity	Clinical and metabolic drawbacks
Local release of cytokines and growth factors:	Need of lactate conversion to bicarbonate in the liver
→ inflammatory state	
→ fibrogenic processes	
→ neo-angiogenesis	Loss of bicarbonate due to its back-diffusion into the dialysate
→ peritoneal fibrosis	
Impairment of	
→ mesothelial cell integrity	Abdominal pain during the inflow of dialysate
→ peritoneal macrophage function	
→ intraperitoneal host defence	
→ membrane permeability	

In summary, bicarbonate seems to be the most suitable buffer for PD solutions to be used in paediatric patients who frequently undergo the short dwell cycles of night APD schedules [31]. However, long term clinical trials would be needed to confirm the impact of bicarbonate-buffered solutions on peritoneal membrane viability.

### Calcium

Commercially available PD solutions contain: (1) 1.75 mmol/l of calcium; since ionized calcium in these solutions is higher than the ionized calcium level normally present in blood, diffusion of calcium from dialysate to blood would lead to a positive calcium balance; (2) 1.25 mmol/l; these solutions are frequently employed with the goal of reducing the risk of hypercalcaemia, especially in children receiving calcium carbonate or calcium acetate as phosphate binders and being treated with vitamin D analogues [31]. Attention should be paid to avoid hypercalcaemia and a high calcium x phosphate product for the potential risk of inducing vascular and soft tissue calcification. Use of non-calcium-containing phosphate binders would be indicated in these cases.

## Prescription for PD regimen

PD prescription should be tailored on the basis of the child’s age, body size, residual renal function (RRF), nutritional intake, and transport capacity of the peritoneal membrane. Moreover, the prescribed PD schedule should be compatible with the psychological and social needs of the patient and family. Technical parameters that should be primarily considered in the prescriptive process are [4, 13, 52]:

1. status of peritoneal membrane transport, evaluated by means of validated functional tests
2. PD solution, selected according to biocompatibility and potential UF capacity
3. fill volume, tailored to the patient’s needs and tolerance
4. exchange dwell time, optimized for small and middle-sized molecule removal and UF

### Prescription of fill volume

Scaling IPV by patient BSA has become a standard in paediatric PD prescription [4, 13, 52]. IPV and the patient’s posture dynamically affect the recruitment of an effective peritoneal membrane area for dialytic exchange, which corresponds to the unrestricted pore area over diffusion distance (A_0_/ΔX) as determined with the three-pore model [21, 23]. Raising IPV from 800 ml/m^2^ BSA to 1,400 ml/m^2^ BSA leads to maximization of peritoneal vascular surface area [23]. On the other hand, excessive IPV may cause patients discomfort, pain, dyspnoea, hydrothorax, hernia, gastroesophageal reflux, and loss of UF due to increased lymphatic drainage. Hydrostatic intraperitoneal pressure (IPP) is a reproducible patient-characteristic parameter, and its measurement helps to evaluate fill volume tolerance in the individual patient [53]. For a patient in a supine position, fill volume leading to an IPP of 18 cm H_2_O is considered to be the maximum tolerable IPV, above which abdominal pain and a decrease in respiratory vital capacity may occur [52]. An IPV of 1,400 ml/m^2^ BSA seems to be the optimum for ensuring optimal recruitment of vascular pore area in children; however, this should be considered as a maximum limit, the safety of which has not been validated in children. In clinical practice, fill volume can be increased in steps up to the limit of 1,400 ml/m^2^ BSA for a night exchange, while the patient is lying down, according to clinical tolerance and IPP measurement [52].

### Prescription of dwell time

Dwell duration should always be determined according to the individual patient’s transport status [4, 13, 52]. Short exchanges lead to satisfactory clearance of small solutes (such as urea) and UF, which can be further enhanced by increasing dialysate glucose concentration. Patients with high rates of transport would benefit from short exchanges, due to dissipation of osmotic gradients by fast glucose absorption. Long exchanges favour the removal of solutes of relatively high molecular weights, such as creatinine and phosphate, but can be associated with impaired UF or even with dialysate reabsorption if glucose-based solutions are being used. An icodextrin-based solution is more appropriate for such long dwells [31, 33].

A potentially useful way to individualize dwell duration in paediatric patients on automated PD (APD) according to peritoneal transport capacity is the calculation of the so-called APEX time. In the PET, the APEX time corresponds to the point at which the D/P urea and D/D0 glucose equilibration curves cross, and should represent the optimal length of APD cycles [54].

The above-mentioned prescription principles should be applied to the delivery of different PD regimens, which will be briefly described [4, 31, 55].

### Continuous ambulatory peritoneal dialysis

CPD was originally performed as CAPD, a continuous regimen that allows complete equilibration of small solutes and a certain removal of middle-sized molecules. This PD modality has the undoubted advantage of ease of use and limited cost of the equipment. CAPD is usually effective in children with RRF, but its decline should be closely monitored. According to the guidelines of the European Committee on adequacy of the pediatric PD prescription [4], initial fill volume can be 600–800 ml/m^2^ during the day and 800–1,000 ml/m^2^ overnight, and can then gradually be increased according to the patient’s tolerance and IPP measurements. An icodextrin-based solution can be used for the prolonged night-time dwell. As a further step, the number of exchanges can be increased from four to five per day. However, if increasing the number of exchanges to obtain adequate UF and solute removal represents an excessive burden upon the families, a shift of the patient to an APD modality should be considered.

### Automated peritoneal dialysis

APD represents the PD modality of choice for paediatric patients, at least in countries that have no cost constraints [2, 3]. Financial and technical problems still represent a limitation to the use of APD for many units in developing countries.

Reasons for the preference of APD are reported in Table 3. APD offers a wide selection of treatment schedules that has in common the use of automated machines for fluid delivery, currently called ‘cyclers’, and the high efficiency obtained through short dwell times, high dialysate flows, and large IPV.

**Table 3:** Reasons for preference of automated peritoneal dialysis (APD) in paediatric patients (*IPP* intraperitoneal pressure, *PM* peritoneal membrane)

Why APD is preferred
Wide range of treatment options
➔ tailoring of APD prescription according to:
- age
- body size
- clinical conditions
- growth-related metabolic needs
- residual renal function
- PM transport status
Large fill volume in the night-time exchanges
➔ recruitment of functional peritoneal surface area
Option of an empty abdomen during the day
- normal IPP (less risk of hernias)
- reduced glucose absorption
- reduced exposure of PM to dialysis fluid
- reduced loss of proteins and amino acids
Psychological and social rehabilitation
➔ reduced impact of treatment on patient/family lifestyle

### Nightly intermittent PD

Nightly intermittent peritoneal dialysis (NIPD) consists of a number of short nocturnal cycles, without a daytime dialysate dwell, and is primarily indicated for patients characterized by a high-transport peritoneal membrane, which allows rapid solute equilibration. The main advantages of a dry abdomen during the day include normal IPP, and the reduction of glucose absorption, of AA and protein loss, and of membrane exposure to glucose. On the other hand, the absence of a daytime dwell is a limitation for solute clearance (especially for middle-sized molecules), and it makes NIPD not suitable for patients with low and low-average peritoneal transport. NIPD is frequently adopted as the first APD regimen for patients with a significant RRF. Then, NIPD prescription can evolve according to clearance and UF requirements, that are mostly dictated by the decline of RRF. NIPD efficiency can be enhanced by increasing dwell volume, total treatment time, and the number of exchanges up to a point beyond which solute clearance and water removal may decrease as the non-dialytic time, corresponding to the fill and drain phases, becomes unacceptably long.

### Tidal PD

With this modality, an initial infusion of solution into the peritoneal cavity is followed by only partial dialysate drainage; thus, an intra-abdominal volume is always left. Drained tidal volume is replaced with fresh dialysis fluid to restore initial IPV, while the entire dialysate volume is drained at the end of the PD session. The amount of ultrafiltrate expected to be generated during each cycle must be estimated and added to the drain volume, to prevent overfilling of the peritoneal cavity. Continuous contact between dialysate and peritoneal membrane maintains a sustained diffusion of solutes, and the efficiency of the dialysis modality is further increased by reducing inflow and outflow dead times, particularly when high dialysate flow rates are used. Tidal PD is also adopted to avoid repeated cycler alarms of low flow rate in case of catheter malfunction, and to reduce pain occurring during the drainage phase. The efficiency of tidal peritoneal dialysis (TPD) is mainly conditioned by total volume of PD fluid delivered and by the individual’s peritoneal transport rate. Patients with high transport rates can reach adequate solute clearances with intermittent nightly TPD, while patients with high average transport rates would benefit from one or more daytime dwells [56, 57]. A further increase of TPD efficiency can be attained by adapting tidal volume to the drainage profile of each patient, thus reducing the fill and drain dead times [58]. In fact, the drainage profile of peritoneal fluid is not linear, since a high flow rate is maintained until the so-called breakpoint, when a critical intraperitoneal volume is reached. Then, the flow rate drops, and, during the final, slow-flow portion of the drainage phase, the peritoneal cavity is almost empty and solute clearance is greatly reduced [59]. Since critical intraperitoneal volume is an individual characteristic, tidal volume can be tailored to the drainage profile of each patient, thus reducing idle time and improving the overall efficiency of the system.

**Fig. 1:**
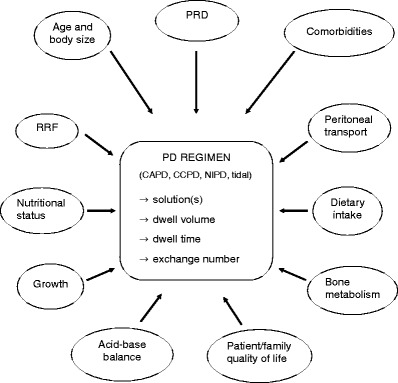
Factors that should be accurately evaluated for each individual patient in the process of peritoneal dialysis (PD) prescription, and PD regimen parameters that have to be defined to achieve the final treatment schedule (*PRD* primary renal disease, *RRF* residual renal function)

### Continuous cycling PD

In CCPD, a fresh exchange of dialysis solution, ranging in volume from 50% to 100% of the fill volume, applied at night, is left in the abdomen at the end of the nocturnal APD session. Daytime exchange dialysate can be drained at bedtime, when the cycler is reconnected, so that the patient’s involvement is reduced to one session for preparation of the equipment and connection to cycler, and one short disconnection in the morning. Over a long daytime exchange, complete equilibration of small-solute concentration between plasma and dialysate is often achieved; moreover, middle-sized uraemia toxins are poorly influenced by short cycles of APD and much more dependent on complete saturation of dialysis solution during long dwell exchanges [60]. Phosphate PD clearance is usually insufficient to obtain a satisfactory control of hyperphosphataemia, and there is a continued need for dietary restriction and phosphate binder administration; however, phosphate removal by PD can be improved by increasing dwell volume and by optimizing exchange duration through the calculation of the so-called phosphate purification dwell time (PPT) from a PET [54].

A continuous PD regimen is recommended when RRF becomes negligible, and it is indicated in patients with high-average rates of peritoneal transport. Icodextrin solution is typically employed for the long daytime exchange [31, 33, 34]. If a further increase in solute clearance and UF is desired, more than one diurnal exchange can be performed, optimizing the length of each dwell according to the patient’s peritoneal transport rate and the type of osmotic agent employed (continuous optimal peritoneal dialysis, COPD) [4, 60].

The delivered dialysis dose should be adjusted and monitored in accordance with the 2006 update of the National Kidney Foundation/Kidney Disease Outcomes Quality Initiative (NKF-KDOQI) clinical practice recommendations for paediatric peritoneal dialysis adequacy (Table 4) [13]. In the absence of definitive results from large randomized controlled studies on the correlation between solute removal and clinical outcome in paediatric patients treated by PD, current clinical opinion supports the recommendation that the target delivered solute clearance should meet or exceed that of the adult standards. In patients with RRF, the contribution of renal and peritoneal clearance can be added for practical reasons. In general, results of the prescribed PD schedule should regularly assessed, taking into account not only numerical targets of small-solute depuration, but also all the parameters involved in the definition of adequacy of dialysis treatment in childhood, such as adequate growth, blood pressure control, and nutritional status; avoidance of hypovolaemia and sodium depletion; adequate psychomotor development [4, 13, 14].

**Table 4:** Solute clearance targets and measurements in children on maintenance peritoneal

Targets and measurements
The minimal delivered dose of total (peritoneal and kidney) small-solute clearance should be a Kt/V_urea_ of at least 1.8/week
Total solute clearance should be measured in a clinically stable patient:
➔ within the first month after dialysis has been initiated
➔ *at least* once every 6 months thereafter (but in patients with residual renal function 24 h urine collection for determination of volume and solute clearance should be obtained at a minimum of every 3 months)
➔ more frequently when clinical events are likely to have resulted in decreased clearance or when new/worsening signs or symptoms of uraemia develop
➔ *at least* 1 month after resolution of an episode of peritonitis
➔ if a patient is not doing well and has no other identifiable cause other than kidney failure
When calculating Kt/V_urea_, V, or total body water, should be estimated by using gender-specific nomograms based upon equations that include the patient’s height and weight [77]

### The use of software programs for APD prescription

In the clinical setting, the selection of a personalized PD schedule, among the wide range of treatment options that are available with APD, can be facilitated by kinetic modelling. Mathematical modelling software programs, that have a specific individual peritoneal function test as data entry, have been developed to calculate kinetic parameters, to simulate the results of APD regimens, and rapidly find the best personalized dialysis schedule, thus omitting the conventional ‘trial and error’ approach [61]. Two of these software programs have been validated in children [9, 22, 62]. The accuracy of these mathematical models in predicting solute removal was good, while UF prediction was less accurate. The limited performance with respect to UF prediction may be related to the inability of kinetic modelling to account for changes in residual dialysate volumes, the marked day-to-day variability of UF, the large variability of daily fluid intake, and the confounding effect of residual diuresis in non-anuric patients [63, 64]. Moreover, mathematical modelling refers to perfect and virtually uneventful APD sessions, with no alarms and no delay in the fill and drain phases; therefore, simulation may, at times, be ‘optimistic’. In conclusion, computer-assisted kinetic models are useful tools for selecting the optimal dose of dialysis for a given patient, but direct measurement of actual solute clearances and UF rate remains mandatory.

## Technical issues

Over the past 15 years, CPD has experienced a great and quite fast evolution, which has been mostly linked to the development of safe and simple-to-use connecting devices, more biocompatible solutions, and new automatic machines for PD delivery (cyclers).

The use of an integrated Y set, double-bag system, with a disinfectant-containing cap, and a ‘flush before fill’ mode, has been associated with a reduction in the incidence of peritonitis episodes due to manual contamination and has contributed to the simplification of PD connecting manoeuvres, thus shortening patient and partner training [55, 65–67].

Modern cyclers for APD are characterized by small size, light weight, and portability, as well as their user-friendly interface, which represent basic requirements for home-based therapy devices [55, 68]. From a technical point, these machines have a great programming flexibility and are equipped with a cassette specific for the tubing set, and automated devices for its connection with the dialysate bags to minimize the risk of operator error and contamination; online warming of dialysate; gravity-assisted pumps for dialysate infusion and drainage; pressure monitors to assess IPP [68].

APD of paediatric patients is performed through a specific paediatric mode of the cycler, which allows accurate delivery of small dialysate volumes (60 ml per exchange, with 10 ml increments), with as limited a recirculation as possible, and drainage of peritoneal effluent at a low flow rate without triggering the alarm.

The technology incorporated in the cyclers has also led to the possibility of treatment prescription and events being memorized on an electronic device, thus providing information on the delivered dialysis dose and UF, on patient’s compliance to APD prescription, and on peritoneal catheter function. For instance, cyclers can record the drainage profile of each exchange, giving graphs of the pattern of catheter flow, and can detect the breakpoint in each cycle, and accordingly adapt the drain profile. This database of therapy information can be downloaded from the memory card of the cycler during the patient’s visits to the dialysis unit, or it can be retrieved via a modem on a regular basis. The telecommunication link between the patient’s cycler at home and a computer at the dialysis unit can improve the care of patients living at distance from the dialysis centre, through the early detection of a series of clinical and technical problems [69]. For instance, these might be represented by an imperceptible, but progressive, decrease in UF rate, or by a prolongation of the drainage phase due to a catheter malfunction that is still too small to trigger cycler alarms. Moreover, the awareness of routine data recording and transmission can help the patient to be more confident of treatment control and help the dialysis doctors and nurses to update PD prescription more rapidly. The tele-dialysis system can also be integrated into video-conferencing equipment to conduct tele-consultations [70, 71]. In the only available paediatric report on this kind of tele-care support, the videophone equipment employed still showed technical limitations and was considered to be not cost-effective [72]; therefore, this technology deserves further evaluation in paediatric home PD.

Further advances in cycler technology will include the ability to optimise PD regimens, by using the recorded information on the patient’s response to a given treatment to suggest an improved schedule, or even to attempt to improve it automatically. As examples, online detection of UF could serve as information for automatic feed-back on the bedside production of the next cycle PD fluid, which will be individualized with respect to osmotic agent, buffer, calcium, and sodium content [68, 73, 74].

## Conclusions

In conclusion, issues that may have a major impact on the ability to individualize PD treatment and to preserve the efficacy of the prescribed regimen over time will be briefly recalled (Fig. 1).

An important step in the process of individualizing PD prescription is represented by the characterization of PM transport capacity, which should be assessed by means of well standardized functional tests that have been validated in paediatric patients.

Fill volume should be scaled to body surface area and adapted to each patient, according to clinical tolerance and IPP measurement, in order to ensure maximum recruitment of peritoneal exchange area.

Children represent a patient category that would greatly benefit from the use of new, more physiological and biocompatible, PD solutions, especially if one considers their long-term dependence on a functioning peritoneal membrane in case of a kidney transplant failure and the fact that, in APD, frequent short cycles continuously expose the peritoneal membrane to a non physiological and bio-incompatible milieu. Combined use of glucose, amino acids, and icodextrin as part of a glucose-sparing APD regimen, together with the adoption of pH-neutral solutions, may represent a strategy that would adequately manage solute removal and UF, while preserving PM integrity over time.

Fluid balance is increasingly recognized as a crucial aspect of the PD patient’s treatment, as the efficiency of water and salt removal is associated with patient outcome, especially in anuric patients, and UF failure is an important cause of failure of the technique [75].

Prospective randomized trials of dialysis adequacy and observational studies in adult patients have confirmed that RRF is a much stronger predictor of patient survival than peritoneal clearance. Therefore, the PD prescription should be aimed to preserve RRF for as long as possible [13, 76], by gradually increasing the dialysis dose in steps, accurately targeting UF rate to maintain the patient’s dry body weight, and using the lowest possible dialysate glucose concentration required to achieve the desired UF volume. Prevention of RRF loss also involves the avoidance of nephrotoxic insults (medications, radiocontrast agents, urinary obstruction and infection) and the use of loop diuretics. The potential role of angiotensin-converting enzyme inhibitors and angiotensin-receptor blockers is worth investigating in interventional outcome studies in children on PD. As RRF declines over time, PD prescription should be adjusted in a timely fashion.

The evolution of APD has been closely linked with the advances in the technology incorporated in the new cyclers that have made APD delivery safer and more efficient. Whether tele-dialysis is able to reduce significantly the need for patient hospitalization or the incidence of technique failure in a population of home APD children should be evaluated in large-scale studies.

The ultimate goal of the whole process of PD modality selection and prescription is to identify, and possibly achieve, the optimal PD dose for each individual patient; this can be regarded as the amount of dialysis above which the additional expected benefit does not justify the increase of the burden on patient and family and of financial costs.

## Questions

(Answers appear following the reference list.)

1. On what anthropometric parameter should fill volume prescription be scaled in paediatric patients ?
  - Weight
  - Height
  - Body surface area
  - Body mass index
2. What is the pH of standard, lactate-based PD solutions?
  - 7.0–7.6
  - 5.5–6.5
  - 4.0–4.5
  - < 4.0
3. Through which chemical mechanism does icodextrin induce fluid removal?
  - Crystalloid osmosis
  - Diffusion
  - Colloid osmosis
  - Hydrostatic pressure
4. Which of the following solutes is more efficiently removed during short dwells?
  - Creatinine
  - Phosphate
  - Beta-2-microglobulin
  - Urea
5. In what prescription parameter do nightly intermittent PD and continuous cycling PD mainly differ from one another?
  - Number of night-time exchanges
  - Dry or wet abdomen during the day
  - Tonicity of dialysate
  - Night-time fill volume
